# ATP7A-Regulated Enzyme Metalation and Trafficking in the Menkes Disease Puzzle

**DOI:** 10.3390/biomedicines9040391

**Published:** 2021-04-06

**Authors:** Nina Horn, Pernilla Wittung-Stafshede

**Affiliations:** 1 John F. Kennedy Institute, 2600 Glostrup, Denmark; 2 Department of Biology and Biological Engineering, Chalmers University of Technology, 41296 Gothenburg, Sweden; pernilla.wittung@chalmers.se

**Keywords:** ATP7A, Menkes disease, symptomatology, copper enzyme, copper trafficking

## Abstract

Copper is vital for numerous cellular functions affecting all tissues and organ systems in the body. The copper pump, ATP7A is critical for whole-body, cellular, and subcellular copper homeostasis, and dysfunction due to genetic defects results in Menkes disease. ATP7A dysfunction leads to copper deficiency in nervous tissue, liver, and blood but accumulation in other tissues. Site-specific cellular deficiencies of copper lead to loss of function of copper-dependent enzymes in all tissues, and the range of Menkes disease pathologies observed can now be explained in full by lack of specific copper enzymes. New pathways involving copper activated lysosomal and steroid sulfatases link patient symptoms usually related to other inborn errors of metabolism to Menkes disease. Additionally, new roles for lysyl oxidase in activation of molecules necessary for the innate immune system, and novel adapter molecules that play roles in ERGIC trafficking of brain receptors and other proteins, are emerging. We here summarize the current knowledge of the roles of copper enzyme function in Menkes disease, with a focus on ATP7A-mediated enzyme metalation in the secretory pathway. By establishing mechanistic relationships between copper-dependent cellular processes and Menkes disease symptoms in patients will not only increase understanding of copper biology but will also allow for the identification of an expanding range of copper-dependent enzymes and pathways. This will raise awareness of rare patient symptoms, and thus aid in early diagnosis of Menkes disease patients.

## 1. ATP7A-Related Copper Disorders

ATP7A-related X-linked genetic disturbances exhibit dysfunction of multiple copper-dependent processes resulting in a broad spectrum of disease phenotypes. Three clinical groups are described: Menkes disease (MNK), occipital horn syndrome (OHS), and X-linked distal spinal muscular atrophy 3 (SMAX3) but overlapping intermediate forms (Table 1) confuse grouping [1,2]. MNK is characterized by neurodegeneration, fair skin, kinky hair, connective tissue abnormalities, and short life span. OHS presents with connective tissue symptoms, develops pathognomonic occipital bony exostosis (horns), and has reduced life expectancy. SMAX3 comprises a yet limited group of adult-onset progressive motor neuron disease, minimal copper disturbance, normal fertility, and long lifespan. Grouping into three phenotypes is arbitrary, and the spectrum is better described as a clinical continuum from severe disease with many affected enzyme systems to very mild affection with few enzyme systems involved. The best nosology should refer to ATP7A-related disturbances as the main pointer [2]. To best explain patients’ symptoms, we have here focused on the severest form, i.e., MNK.

The basic defect in MNK is deficient copper transfer to secretory pathway, the intracellular sorting station for proteins, which affects metalation and trafficking of copper-dependent enzymes and diminishes copper extrusion from cells. Insufficient functional ATP7A results in abnormal body copper distribution with high values in tissues, but severe lack in blood, brain, and liver [1]. Current understanding of copper dependent processes does not account for all neurological symptoms [3], and a broad update is needed. Substrates are included with each enzyme to better define key symptoms in MNK.

Clinical features found in MNK patients can be explained by deficiencies of copper-dependent enzymes, amine oxidases (biodegradation of histamine and polyamines), lysyl oxidases (cross-linking of elastin, collagen, and collectin), cytochrome c oxidase (energy formation), peptidyl α-amidating enzyme (activation of neurohormones and neuropeptides), dopamine β-hydroxylase (catecholamine production), tyrosinase (pigment formation and free radical defense), superoxide dismutase (free radical detoxification), and ferroxidase (iron mobilization and free radical defense). In addition, newly discovered copper-activated enzymes, including several lysosomal and steroid sulfatases, provide further insight into MNK pathophysiology [4] and will be discussed herein.

## 2. Copper Enzymes

Copper serves as redox cofactor in a wide range of reactions including electron transfer, oxidation, reduction, and disproportionation [4] (Table 2). Copper is regulated by redox shifts at several cellular and subcellular membranes and is further implicated in redox shifts of iron. In addition, copper acts as allosteric regulator at binding sites spatially apart from catalytic centers (Table 2). In enzymes, copper acts as integral electron modulator, but roles in formation and coupling of cofactors are important for understanding MNK clinical spectrum [4] and copper chaperone activation of enzymes without catalytic copper sites is emerging [5]. Copper enzymes contain active centers shuttling electrons from one molecule to another, and copper changes reversibly between oxidation states during catalytic cycles. Nature uses a variety of copper centers to facilitate electron transfer, and enzymes are grouped accordingly. Often catalytic metal sites are made up of metal coordinating residues located far apart in primary structure, but in the folded structure, they create a cluster of closely spaced amino acids forming metal-binding sites. The protein’s tertiary structure is often stabilized by coordination of the metal [6,7].

Copper enzymes are widely distributed both intracellularly and extracellularly. Intracellular copper enzymes are located in subcellular compartments and organelles such as cytoplasm, secretory pathway, peroxisomes, nucleus, and mitochondria, and metalation occurs at different sites. It is an important question to ask when and where the polypeptide meets the metal for copper-dependent enzymes. Newly synthesized apoenzymes are directed to the secretory pathway, and during passage from endoplasmic reticulum (ER) to *trans*-Golgi Network (TGN), maturation, assembly, glycosylation, and metalation occur. Copper transfer uses different mechanisms involving specific copper chaperones and combine metal coordination chemistry with protein-protein interactions for donor-acceptor docking. Specific copper chaperones for all copper-dependent enzymes have not been identified, and additional ones are likely to be discovered in the future. Cellular copper redox states and concentrations are strictly controlled, and free copper ions are kept at low, non-toxic levels. Within secretory pathway pH is gradually shifted towards an acidic environment and from oxidizing to reducing milieu changing the strength of copper binding [8,9]. Centers with tight metal coordination are preserved during secretory passage, while centers with low avidity can lose copper, and chaperones may be needed to protect their lability. Several copper enzymes possess labile copper sites, such as SOD1, SOD3, DBH, PAM, and TYR, while CP and HEPH have more stable copper sites. 

This review centers on function and biogenesis of copper-dependent enzymes, which are activated/matured by copper loading at various sites in cells, which relates to protein trafficking. Cellular copper homeostasis is extensively covered in other reviews, e.g., [10,11,12], and will not be discussed here unless pertinent for understanding activation of copper enzymes.

## 3. Copper-Dependent ATPases

Copper-transporting ATPases, α (ATP7A) and β (ATP7B), are homologous P-type ATPases utilizing energy for pumping copper across a membrane. They are ion gated channels crucial for cellular and whole-body copper homeostasis. They have similar but distinct functions, and supplement and complement each other to fine tune equilibrium by transporting copper in different tissues and by coordinating activity in specific cells [11,13]. Both enzymes pump copper from cytoplasm into compartments with higher copper concentration [14]. ATP7A moves copper out of cytosol and across the basolateral membrane in extra-hepatic tissues [15], while ATP7B moves copper out of cytosol and across the apical membrane in liver, brain, and kidney [11,16]. ATP7A controls transport across the gut mucosa and the blood–brain barrier (BBB).

ATP7A and ATP7B are multi-domain enzymes that undergo profound changes during pumping [17]. They share highly conserved domain structure and basic mechanism with other P-type ATPases. Eight membrane-spanning helices constitute a pore-forming transmembrane domain for copper translocation. The channel is linked to three cytoplasmic ATP hydrolytic domains plus six metal binding domains (MBD) with copper-specific motifs (GMXCXXC) [18]. MBD’s initiate pumping through ATOX1 copper activation [11]. Each MBD possesses a compact fold linked by a flexible loop, enabling independent and cooperative action [19]. During pumping conformation undergoes a flip-flop movement with sequential changes allowing unidirectional transfer of copper from the entry site, through the channel by two embedded sites, and release from an exit site. A kinked transmembrane segment at the cytosolic interface forms an electronegative platform for electrostatic ATOX1 docking, initiating opening of the entry gate [11,20].

Pump domain interactions depend on conformation and position during the catalytic cycle, and energy for pumping stems from ATP-dependent transient phosphorylation of the cytoplasmic part [13]. Disruption of the cycle at any point reduces copper transfer.

Regulatory mechanisms are slowly unraveled. ATP7A/B pumping activity is via MBD’s controlled by copper. Docking of ATOX1 on the kinked platform, filling and packing of MBD’s serve as metal sensor besides allosteric regulation. Exact molecular mechanisms that modulate ATP7A/B activity still remain unclear [21].

ATP7A/B trafficking is copper regulated. At basic homeostatic levels copper is pumped into the secretory pathway, but at high levels the pumps relocate to excrete surplus. At low copper, a high free GSH pool secure glutathionylation of MBD’s and retention, while high copper results in low glutathionylation and trafficking [22,23]. ATP7A/B contain a histidine and methionine rich lumenal loop located between TM1 and TM2 that may function as ER retention signal [20,24,25,26]. Ca-pumps possess similar regulatory motifs at corresponding locations to secure Ca-guided ER retention [27]. The ATP7A loop motif may act as copper donor in metalation of certain enzymes [28]. ATP7A and ATP7B show distinct copper transport kinetics, where ATP7A is faster than ATP7B [11,29], but underlying reasons for differences are not clear [21].

### 3.1. Copper-Transporting ATPase 1 (ATP7A)

ATP7A regulates tissue copper levels and is expressed in most tissues except postnatal liver. At basal levels ATP7A transports copper into lumen of secretory pathway to load secreted and vesicular copper enzymes. At standard tissue culture conditions ATP7A reside in TGN and when exposed to excess copper, the pump relocates to the plasma membrane to export copper [30]. Some enzymes require metalation in ER, and removal of TGN signal by skipping of the alternatively spliced exon 10 retains the protein in ER [20,24] and may be of functional significance. ATP7A is rate limiting in gut uptake and import to the brain.

ATP7A interacts with a range of adaptor molecules, some affect nerve development [31]. ATP7A contains several N-glycosylation sites [32] and need ERGIC trafficking by a carbohydrate-recognition domain (CRD) and an adaptor complex like LMAN1 [33].

### 3.2. Copper-Transporting ATPase 2 (ATP7B)

ATP7B regulates whole-body copper homeostasis by excreting surplus into bile [34]. ATP7B is expressed in numerous tissues, and plays a role in copper regulation in liver, brain, placenta, and kidneys [4]. ATP7B supplies copper in liver to ceruloplasmin, and clotting factors V and VIII. Gene defects cause Wilson disease (WND) with copper accumulation in liver and brain.

ATP7B is not N-glycosylated [32] and not sugar sorted to the apical canalicular membrane but uses a subset of secretory lysosomes. Trafficking is directed by a motif of aromatic amino acids between MBD4 and MBD5 with loose copper binding [15,35] requiring an acidic milieu to secure high free copper for activation. Consistently ATP7B uses an acidic lysosomal pool for excretion. Inactivation of the trafficking signal directs ATP7B to the sinusoidal membrane [35] to mobilize hepatic stores into circulation [36]. In the brain, ATP7B likely traffics by other mechanisms, though not described. ATP7B is also regulated by alternative splicing of the loop motif [13,37].

## 4. Redox Shifting Enzymes

Intracellularly cupro ions, Cu(I) predominate, while higher extracellular oxidation potential results in cupric ions, Cu(II) [11,38]. A redox shift is needed at copper uptake and export, and organelle membranes likely also require copper redox shifts for transfer. This mimics conclusions for iron transport [39] and at sites the two metals share redox enzymes [40,41]. Copper and iron are reduced at plasma membranes by a heme reductase [42]. Before release iron uses a multicopper oxidase also having copper oxidizing capacity [43]. Iron has higher reduction potential than copper, while copper is superior in oxidative reactions [44,45]. Iron prosthetic groups are involved in a broad range of biological processes. Iron utility depends on careful control of redox state, and specific redox enzymes are found widespread also at subcellular levels. Iron–copper interactions have emerged as crucial, and copper is critical for normal handling of iron [46].

Mitochondria have significant iron and copper stores, securing biogenesis of two iron prosthetic groups, heme and iron-sulfur (Fe-S). Heme is tightly interconnected with copper metabolism and dependent on Fe-S availability [47]. Fe-S clusters are found at several subcellular sites including mitochondrial respiratory chain. Fe-S biogenesis is complex involving numerous steps, some occur in mitochondrial matrix, others in cytoplasm. Fe-S biosynthesis is interconnected with heme biosynthesis [48]. Iron trafficking is not well understood [49], and redox changes critical in organelle metal homeostasis are less known. Copper deficiency leads to low heme-iron which in turn gives insufficiency of enzymes needed for mitochondrial iron membrane translocation [50]. Deficient heme affects copper through dysfunction of membrane uptake, conferring a gatekeeping role for copper in translocation of both iron and copper.

### 4.1. Heme Copper Reductases

Six-transmembrane epithelial antigen of prostate (STEAP) comprises a family of metalloreductases with ability to reduce iron and copper [51]. STEAP4 shows physiological Km values for both metals [52]. Reducing sites use heme and cofactors like NAD, NAD(P)H, and ascorbate as electron donor [53,54]. The STEAP family is widely expressed [51], but tissue-specific expressions suggest distinct roles [53]. STEAP proteins locate at plasma membrane for copper and iron uptake but are also implicated in trafficking by modulating redox states in endocytotic and secretory pathway, and in mitochondria. STEAP1 acts at tight junctions, gap junctions, and cellular adhesion, and is hormone regulated [53]. STEAP2 regulates iron and copper absorption in gastrointestinal tract [53] and flux across BBB [55]. STEAP2 is expressed in most tissues primarily at plasma membrane and Golgi complex, possibly regulating metal availability in secretory pathway [53]. STEAP3 is an endosomal reductase required for efficient iron uptake into erythroid precursor cells. STEAP3 is highly expressed in liver, placenta, and bone marrow [53], and is located at plasma membrane, near nucleus, and in vesicular tubular structures [53]. STEAP4 is ubiquitously expressed at plasma membrane, ER, and TGN, suggesting a trafficking role [53,56]. STEAP4 also localizes at early endosomes and mitochondria [57,58], and splice variants localize to nucleus [59].

Cytochrome B reductase 1 (CYBRD1) is a di-heme reductase with iron and copper reducing capacity located at intestinal brush border to reduce iron and copper for mucosal uptake [60]. It may act also as a reductase in airway epithelium. CYBRD1 reduces iron and copper, uses ascorbate, and possesses 6TM, hence naturally grouped among STEAP.

### 4.2. Multicopper Oxidases

Multicopper oxidases (blue copper oxidases) contain six copper in two complex sites with different redox properties, oxidizing sequentially without formation of free radicals. Cupro and ferro ions are strong pro-oxidants, and multicopper oxidases scavenge radicals by preventing Fenton chemistry [11,43].

#### 4.2.1. Ceruloplasmin (CP) 

Ceruloplasmin (CP) is a major redox buffer in blood converting highly toxic ferro ions to less toxic ferri ions. CP mobilizes iron from stores in liver and other tissues, and copper by securing oxidation state [11,61], not by moving copper from one binding site to another; CP is not part of the exchangeable copper pool. Fet3p, a yeast homologue, exhibits cuproxidase activity at same site having ferroxidase activity [61]. CP is synthesized in liver, but a glycosylphosphatidylinositol-linked form (GPI-CP) is found in astrocytes and choroid plexus [62,63]. GPI is attached in ER and serves as O-glycosylation signal for trafficking to the apical membrane [62,64]. CP is ascorbate dependent and has two deeply embedded catalytic centers [6,65,66], copper loaded in liver by ATP7B, and released to circulation from the sinusoidal membrane. Apo-CP passes to Golgi though exact metalation site is not identified [65,67]. The apoform is secreted into blood, but rapidly degraded [68]. It lacks ferroxidase activity [65], and cannot be copper activated later [66], underlining that CP is not a transport form for tissue copper exchange [11]. Aceruloplasminemia leads to iron accumulation in brain and Parkinson-like ataxia and progressive dementia [69]. In ATP7A-related disturbances, CP metalation is not affected, but low hepatic copper results in low plasma holoenzyme activity. Ferroxidase activity is also low in other tissues, especially brain GPI-CP. Low plasma CP is well-documented in MNK, and some patients show moderate hypochromic anemia, and iron accumulates in kidney and nerve tissue [70]. Poor glycosylation may contribute to poor hepatic secretion, and MNK plasma CP is lower (half) than nutritional copper deficiency and WND [71]. Low ceruloplasmin is a well-established marker for MNK, OHS, and intermediate forms, though less depressed in OHS; but it is not applicable for diagnosis of SMAX3 [2].

#### 4.2.2. Hephaestin (HEPH)

Hephaestin (HEPH) is a membrane-anchored homologue oxidizing iron before release with main role in gut and BBB [64]. Copper oxidizing capacity is likely [11]. The iron metabolome contains large functional redundancy and potentially CP and HEPH can substitute for each other [72], and CP may in absence of HEPH promote iron efflux from enterocytes [73].

HEPH and CP both appear critical for CNS-iron homeostasis [74], and HEPH is abundantly expressed in neurons [75]. CP and HEPH are co-expressed in retina [76] and combined loss lead to age-related macular degeneration (AMD) [72]. Double-knockout *Heph* and *Cp* mice, but neither alone, lead to kidney iron deposition and toxicity [77], and iron accumulation in liver, brain, pancreas, and adipocytes [74,78,79], with significant deficiencies in serum and neurons [80]. Knockout mice exhibit neurodegeneration and retinal degeneration similar to aceruloplasminemic patients. HEPH sites have similar architecture as CP but are loaded by ATP7A [43]. The apoform is rapidly degraded, and by analogy cannot be loaded after biosynthesis [81]. HEPH is regulated by copper and iron; iron induces translocation from intracellular sites to basolateral membrane [82]. HEPH is ubiquitously expressed, most strongly in small intestine followed by kidney [43]. HEPH is N-glycosylated [81], sorted and anchored to vesicles at basolateral membrane [64,81,83].

#### 4.2.3. Hephaestin-Like Protein 1 (HEPHL1)

Hephaestin-like protein 1 (HEPHL1) is a membrane-bound homologue (zyklopen) with similar oxidation functions [84]. Immunostaining shows expression in brain, kidney, testes, and retina, but not liver and intestine [84]. Further expressed in placenta, reproductive tract, and mammary glands and suggested to act in placental iron transport [84]. HEPHL1 requires copper for stability [84], but is less investigated [41,43,64]. Molecular modeling on CP crystal structure indicates preserved HEPHL1 copper sites [84], and by analogy likely involved in copper mobilization. Recent proof is gained by a compound heterozygous patient with abnormal hair, joint laxity, and developmental delay (HJDD) [85]. Hair changes (pili torti and trichorrhexis nodosa) are similar to MNK, and muscular affection similar to SMAX3 [85]. LOX deficiency explains connective tissue involvement and indicates disturbed copper metabolism. HEPHL1 likely acts as redox chaperone in LOX cofactor formation and metalation. Lack of copper affects N-linked glycosylation sites, but none of the O-linked, indicating that copper is loaded before N-glycosylation [85].

#### 4.2.4. Factor V+VIII Clotting Factors

Factor V+VIII clotting factors belong to blue copper oxidases with complex catalytic sites requiring ascorbate as cofactor [86,87]. Both are glycoproteins loaded by ATP7B before secretion to circulation. Within ER, FV and FVIII are guided by mannose binding lectin 1 (LMAN1) for trafficking and secretion [86,88,89]. LMAN1 receptor complex defects cause combined FV+VIII deficiency and mild coagulation disorder [90], underlining importance of mannose binding lectins in glycosylated protein trafficking. LMAN1 is a collectin requiring LOX activation, and in MNK low hepatic copper can affect maturation and trafficking leading to mild clotting deficiency [91].

### 4.3. Cytochrome c Oxidase

Cytochrome c oxidase (COX) uses copper and heme for reduction of oxygen, making mitochondrial copper and iron homeostasis indispensable for life. COX is the terminal oxidase of the electron transport chain comprising four complexes (numbered I, II, III, and IV), each with increasing reduction potential. The inner membrane contains respiratory complexes and copper chaperones; inter-membrane space (IMS) contains Cu/Zn superoxide dismutase 1 and soluble copper chaperones; matrix stores a mobilizable copper pool [92,93]. Copper delivery to COX requires an elaborate machinery of cytoplasmic guiding molecules, outer and inner membrane translocators, and embedded membrane chaperones [94]. Soluble and membrane anchored copper chaperones take different routes: (1) direct uptake via outer membrane into IMS using the redox import machinery [93], and (2) via matrix and redirection. Uptake of complex IV assembly factors is covered in reviews [94,95,96]. Matrix copper is routed to IMS for metalation and activation of enzymes and chaperones. Knowledge about copper exchange between mitochondrial compartments with different redox potentials is limited [97,98]. ATP7A dysfunction affects mitochondrial redox balance [99], and in MNK with low copper in liver and brain, COX deficiency becomes severe. COX is a multimeric complex containing two catalytic copper centers, CuA/heme and CuB, requiring assembly of numerous subunits. Formation of catalytic sites takes place within mitochondria and requires uptake of copper (and iron) into IMS from matrix pools, making insertion after biosynthesis unlikely. Assembly is complicated and assisted by several factors and copper chaperones [93,94,95,96]. The COX assembly will not be covered in detail. However, SCO1, SCO2, and COA6 are inner membrane embedded thiol-disulfide oxidorectases utilizing copper to modulate redox state, before delivery to CuA and CuB [100,101,102]. These copper chaperones are yet examples of redox-dependent copper transfer.

Respiratory chain secures cellular energy production, and disruption affects high energy-demanding tissues like CNS, liver, heart, and skeletal muscles. MNK shows numerous clinical signs of compromised mitochondrial activity [103,104,105]. Patients are hypotonic and floppy, often leading to suspicion of mitochondrial disturbance, and milder cases show myopathy. High lactate in blood or cerebrospinal fluid further strengthens a suspicion. Late disease stages develop deficiency of several respiratory chain complexes [105,106], and muscle ragged red fibers, a sign of mitochondrial dysfunction [103,106].

## 5. Copper-Catalyzed Cofactor Containing Enzymes

This enzyme group needs copper for cofactor activation. Each subclass has its unique activation, some are formed using copper during enzyme biogenesis, while others are attached to holoenzymes by a copper dependent process. SUMF1 dependent enzymes comprise a large new group of mainly lysosomal enzymes only recently joined with copper homeostasis

### 5.1. Copper Quinone Amine Oxidases

Copper quinone amine oxidases contain two subgroups, lysyl oxidases and copper amine oxidases, based on their specific internal cofactors that are formed post-translationally [107,108]. Copper-dependent monoamine oxidases are covered separately below, as they do not use an internal copper cofactor for catalysis. Notably, MAO A and B are not copper catalyzed.

#### 5.1.1. Lysyl Oxidases (LOX)

Lysyl oxidases (LOX) comprise amine oxidases initiating extracellular matrix (ECM) formation by catalyzing oxidative deamination of an epsilon-amino group in lysine and hydroxylysine residues in first step of elastin and collagen cross-linking [108]. Importantly LOX cross-links a variety of triple helical proteins including collectins, significantly expanding LOX functions [109].

All members share a highly conserved catalytic site made up of three His [110] in close proximity to a cofactor created by copper catalyzed cross-linking of two internal amino acids [107,111]. Formation of the internal cofactor, LQT is described as an autocatalytic process, but new evidence points to a need for a redox process [85]. Copper LOX metalation occurs before N-glycosylation [85], and failure to acidify Golgi affects glycosylation resulting in cutis laxa [112,113].

Collagens (COL) and elastin (ELN) provide stability and connectivity among tissues and organs, and fiber building by trimerization and intra- and intermolecular cross-linking is crucial. Other components of ECM, mucopolysaccharides and mucolipids interact with fibers to form connective tissue. Lack of fiber tensile strength affects flexibility and integrity, and results in connective tissue disorders. Tissues affected are bones, tendons, ligaments, joints, muscles, blood vessels, heart, and eyes, with symptoms spanning from osteoporosis, loose joints, lung emphysema, aneurysms, glaucoma to pelvic organ prolapse or rupture [114]. New collectin symptoms are emerging. Numerous COL proteins exist, but type I is most abundant. Clinical defects span from osteogenesis imperfecta to osteoporosis and Ehlers-Danlos syndrome. ELN provides flexibility to fibers that rapidly expand and return to original shape. Deficiency results in joint laxity, wrinkling of skin, aneurysms and emphysema. Gastrointestinal and bladder diverticulae are common. Gly-X-Y triple repeats, hallmarks of COL are versatile and widespread in proteins adapted to a range of functions [115], and an increasing number of proteins with COL-like domains are identified [109]. Trimerization is crucial and needs LOX [115,116,117].

Amongst triple repeat proteins is complement Q1 (C1q), first member of the complement-cascade. Dysregulation of C1q is characterized by recurrent skin lesions, susceptibility to infections, increased risk of autoimmune disease, and chronic kidney disease [118,119]. C1q belongs to collectins [109,120] comprising a superfamily of lectins with a COL-like stretch fused with CRD. Collectins are present in plasma and on cell surfaces acting in first line of defense [109]. Lung surfactant collectins lubricate alveoli, besides being acute phase reactants.

A well described CRD receptor protein is mannose-binding lectin (lectin mannose binding 1) (LMAN1) that triggers the lectin pathway of complement activation. LMAN1 (often named ERGIC-53) is membrane bound and cycles between ER, ERGIC, and cis-Golgi [121,122,123]. LMAN1 and related proteins form complexes with lectin chaperones and is a rate-limiting step in maturation of secreted glycoproteins [89]. Compromised glycoprotein trafficking leads to incorrect localization, and mutations in LMAN1 receptor complex lead to combined deficiency of FV+VIII and hepatic accumulation of α-1-antitrypsin [90]. LMAN1 deficiency leads to susceptibility to meningitis and infections of upper respiratory tract, and as a trafficking factor for neuroreceptors will lead to CNS dysfunction [124,125]. More LMAN1 substrates are being identified also affecting immunobiology [123].

Numerous effector proteins exist, we include only a few here that are most important for MNK. Collagen-like tail of endplate acetylcholinesterase (COLQ) is acetylcholinesterase with a triple-helical membrane anchor to rapidly regulate muscle activation. If deficient, neuromuscular signaling causes muscle weakness [126]. Clinical signs are muscle fatiguability affecting limb muscles, ocular muscles (ptosis and ophthalmoplegia), and facial and mouth musculature (poor sucking and swallowing) as seen in MNK. Collagen and calcium binding EGF domains 1 (CCBE1) protein is important for lymphatic vessel formation, and deficiency results in lymphedema, and mutations in CCBE1 causes Hennekam syndrome [127]. MNK often encounter severe puffiness of face and feet, and a dough-like skin. Collectin defects are accompanied by susceptibility to infections [109], and distinctive facial features also seen in MNK, including widely spaced eyes (hypertelorism), narrowing of eye opening (blepharophimosis), droopy eyelids (ptosis), and high arched (cupid) eyebrows [128,129].

The LOX family is important in relation to MNK and contains five members, lysyl oxidase (LOX) and four lysyl oxidase-like (LOXL) enzymes working on different biological substrates [108,130,131]. LOX is secreted and cross-links ELN and COL. The proenzyme is activated attached to its extracellular substrates [131]. LOX plays a role in aortic wall formation, and deficiency predisposes to aortic aneurisms and dissections [132,133], a prevalent cause of death in MNK. Copper is loaded during Golgi passage, before final N-glycosylation, and a redox step is required [85]. LOX structure has been determined by homology modeling using LOXL2 [134] demonstrating copper coordination by three His and LQT oxygen [110] explaining a redox need in formation of the active center [85]. LOXL1 preferably cross-links ELN [135] and is linked to glaucoma, cataract [136,137], and lens zonule weakness eventually leading to lens subluxation. It is a major risk factor for pseudoexfoliation syndrome. Preferred substrate for LOXL2 is Type IV COL [138], a basement membrane component scaffolding other ECM molecules [130,139]. LOXL2 oxidizes histone and localizes in pericentromeric region [140]. Defects are associated with diseases of muscle, neural, ocular, cutaneous, vascular, lung and kidney tissues [141,142]. Substrates of LOXL3 are not clearly defined, but defects have been associated with early onset myopia [143] and Stickler syndrome that is a group of connective tissue defects with variable facial features, eye abnormalities, hearing loss, and joint problems [144]. LOXL3 localizes in nucleus and is involved in histone biology [145]. LOXL4 is expressed in cartilage and many tissues, the highest levels found in skeletal muscle, testis, and pancreas.

MNK shows numerous LOX and LOXL deficiency symptoms [146,147], and non-accidental injury (NAI) is often suspected [148,149,150], and an important differential diagnosis. LOX deficiency is also well established in OHS [1].

#### 5.1.2. Copper-Containing Amine Oxidases (AOC)

Copper-containing amine oxidases (AOC) comprise a family of both diamine (DAO) and polyamine (PAO) oxidases. AOC participates together with numerous transporters and enzymes to precisely regulate polyamine pathways in CNS and periphery. Functions regulated are wakefulness, inflammation, and neurotransmitter release [108]. In addition, a catalytic role, copper is required for biogenesis of the internal topaquinone cofactor (TPQ) [108]. By analogy, a copper oxidase is likely required for formation of the active catalytic site where copper bridges three His and oxygen of TPQ. Histamine (monoamine), putrescine (diamine), spermidine (triamine), and spermine (tetramine) are ubiquitous AOC regulated amines, involved in proliferation, differentiation, and apoptosis, and in modulation of neurotransmitter receptors [151]. Polyamines play important roles in rapidly dividing cells like immune cells and enterocytes, and in regulation of membrane potentials in excitable tissues. Interactions with ligand-gated ion channels and tight-junctions are emerging as crucial polyamine regulated functions [151,152].

Polyamines are stable compounds present in mM amounts as free (minor), bound, and conjugated forms. Polyamine homeostasis is precisely regulated by de novo synthesis, extracellular catabolic control by AOC, intracellular regulatory feed-back loops, and membrane transfer by solute carriers (SLC) to balance intracellular and extracellular pools [153]. Physiological functions of charged polycations are not fully understood [153]. We focus on accumulation of specific polyamines, the result of deficient AOC catabolic control.

Histamine is the best studied amine, expressed at numerous sites including mast cells, gastrointestinal tract, and neurons [154]. Histamine regulates gastric acid secretion and CNS neurotransmission, in addition to a range of inflammatory reactions [155,156] mediated by specific histamine receptors [156,157].

Abnormal spermidine catabolism results in skin (ichthyosis), hair (alopecia), and eye (conjunctivitis) problems [158,159]. Allergic reactions (atopy), light intolerance (photophobia), and cornea inflammation (keratitis) may occur. High polyamine levels trigger persistent diarrhea with gastrointestinal polyps [154]. All these symptoms are found in MNK patients.

Humans have three AOC: AOC1, diamine oxidase, histaminase, or amiloride-binding protein 1 (ABP1), is mainly expressed in kidney, placenta, intestine, thymus, and seminal vesicles [108], and released at plasma membranes in response to external stimuli. AOC2, or retina-specific amine oxidase, is expressed on cell surfaces in many tissues with a particular high expression in retina [160]. AOC3, also named vascular adhesion protein 1 (VAP1), is widely distributed with highest expression in peripheral lymph nodes, hepatic endothelia, appendix, lung, and small intestine [108]. AOC3 has been implicated in lung inflammation, asthma, psoriasis, and vascular stroke [108]. Expression is also high in white fat tissue where it may be implicated in adipocyte differentiation and metabolism [161].

### 5.2. Formylglycine Activated Sulfatases

Sulfation/desulfation regulate numerous pathways, and sulfatases are responsible for break down and recycling of both complex sulfated sugars and hormones [162,163]. Sulfatases share a post-translationally formed internal cofactor, FGly essential for activity [164,165]. Cofactor generation requires sulfatase-modifying factor 1 (SUMF1) or formylglycine generating enzyme (FGE) [163,166]. SUMF1 oxidises cysteine in target enzymes using a highly conserved sequence, CXPSR [166,167], and recently copper was found to be required [167]. SUMF1 is an ER located soluble glycoprotein acting on native sulfatase polypeptides [168]. ER resident SUMF2 [169,170], a non-copper binding paralog acts as chaperone and retains SUMF1 by heterodimerization while activating sulfatases [171]. SUMF1 interacts with numerous trafficking factors including LMAN1, and lack of activation and trafficking leads to proteasomal degradation of SUMF1 [172]. Sulfatases localize to subcellular sites such as lysosomes, Golgi, and ER [170], where they break-down complex mucopolysaccharides, mucolipids, and steroid hormones. Lysosomal glycosaminoglycan (GAG) sulfatases comprise a major group [173]. GAGs are complex sugar polymers and important components of bone and cartilage, joint lubricants, and cell surface initiating growth factor activity and first line of defense against microorganisms. Recycling of GAGs starts by removal of sulfated groups and defective recycling results in GAG accumulation. Deficiencies present as mimicry of mucopolysaccharidoses (MPS) and mucolipidoses (MLP) affecting multiple organ systems [162,170,173]. Sulfatation/desulfatation are crucial for cartilage formation, and defects are often accompanied by bone dysplasias [173].

Most steroids, e.g., cholesterol, pregnenolone, and estrone, are sulfated after biosynthesis [162], and sulfatation is vital for endocrine function. Cholesterol is crucial for neurotransmission, myelination, and synaptogenesis [174], and desulfatation provides a copper link. Dysregulation is associated with numerous pathologies, including faulty regulation of GABA receptor function [175,176]. Niemann-Pick C disease may be accompanied by copper disturbance likely secondary to poor steroid sulfatase activity and disrupted trafficking of cholesterol [177].

Combined impairment of all sulfatases, multiple sulfatase deficiency (MSD), are clinically heterogeneous disorders caused by mutations in SUMF1 or SUMF2 [169,170]. Symptoms present features of metachromatic leukodystrophy, mucopolysaccharidosis, chondrodysplasia punctata, hydrocephalus, ichthyosis, neurological deterioration, and developmental delay.

ATP7A-related disturbances may mimic MSD and present with overlapping clinical features from a complex interplay between SUMF1 and the LOX family. Sulfated molecules build up in lysosomes, resulting in necrosis and metachromasia, a sign noted early in MNK [178], but forgotten when the copper disturbance was discovered. Morphologic changes with vacuoles in myeloid cells, termed Alder Reilly anomaly are seen in patients with mucopolysaccharidoses (MPS) and have also been reported in MNK [179,180]. Skin problems in MNK may be related to deficient steroid sulfatase (ichthyosis) [181,182] also affecting keratinocyte biogenesis and hair development [183]. Build-up of cholesterol sulfate in the outermost layer of epidermis causes hyperkeratosis with scaling [184].

## 6. Copper-Dependent Mono-Amine Oxidases

Copper monooxygenases catalyze reactions in catecholamine and hormone pathways. The group consists of four enzymes that are free or membrane attached within vesicles of same embryonic origin: adrenal chromaffin vesicles (DBH), synaptic vesicles of the sympathetic nervous system (DBH), secretory vesicles of the pituitary gland (PAM), and melanocytes in periphery and CNS (TYR). Enzymes travel to their final destination, but trafficking is not completely understood and depend on metalation and N-glycosylation. Crystal structure of DBH shows two copper sites, one (CuH) coordinated by three His, the other (CuM) by two His and one Met [185]. Topology is similar to PAM, and also shows likeness to TYR [185,186]. All sites have similar copper avidity [28].

### 6.1. Dopamine β-Hydroxylase (DBH)

Dopamine β-hydroxylase (DBH) is an ascorbate-dependent monooxygenase converting dopamine (DA) to norepinephrine (NE) [187,188]. DBH localizes in synaptic vesicles in noradrenergic and adrenergic nerve terminals of central and peripheral nervous system, as well as adrenal medulla [189]. DBH is targeted to secretory granules by ER glycosylation [185].

DBH contains three N-glycosylation sites [185], and may show trafficking problems [190], and misfolding is suggested to cause DBH deficiency [190]. ATP7A supplies copper to DBH both centrally and in the periphery [191,192,193] and is needed during formation and maturation of the holoenzyme, though copper can likely be loaded later. Met-His-rich lumenal loop of ATP7A can experimentally transfer copper to DBH [28]. NE controls mood, attention, and overall arousal, as well as stress, learning, and memory [185], and the adrenal system is important in maintaining blood pressure, glucose, and sodium levels [194]. Congenital NE deficiency shows profound autonomic failure [188], and perinatal period may be complicated by vomiting, dehydration, hypotension, hypothermia, and severe hypoglycemia all seen in early MNK. Later symptoms are dizziness upon standing (orthostatic hypotension), blurred vision, and difficulty in exercising. Other symptoms are droopy eyelids (ptosis), nasal congestion, muscle pain, and weakness, symptoms well recognized in MNK. DA/NE ratio is increased in plasma and CSF, and dopaminergic imbalance is an early discriminatory marker for MNK [195], and milder forms also show abnormal values [2].

### 6.2. Peptidyl α-Amidating Enzyme (PAM)

Peptidyl α-amidating enzyme (PAM) activates a vast amount of neuroendocrine hormones involved in regulation of numerous processes. PAM is a bifunctional enzyme, consisting of two distinct catalytic domains working sequentially, peptidylglycine α-hydroxylating monooxygenase (PHM) and peptidyl–α-hydroxyglycine α-amidating lyase (PAL). Copper containing PHM catalyzes hydroxylation of a glycine, subsequently cleaved by PAL to generate C-terminal amidation in activated peptide hormones. PHM has copper-binding sites similar to DBH and also requires ascorbate as cofactor [185,186]. Lack of metalation does not alter passage through secretory pathway, and the apoenzyme is not degraded [196], though not directed to correct vesicular location [186]. Copper required for enzyme activity is not tightly bound [7] and can be lost, but secreted apoenzyme can be activated [197]. This likely also apply for trafficking and metalation of related enzymes, DBH and TYR. The first luminal loop of ATP7A involved in release of copper contains an amino acid stretch rich in His and Met acting as potential copper donor for metalation of PAM in secretory pathway [186]. Functionally PAM and DBH overlap, and neuropeptides and neurotransmitters participate in a large number of processes related to feeding and body weight, fluid balance, pain, anxiety, memory, circadian rhythms, and reward [186,198]. PAM is essential for activation of numerous neuroendocrine peptide hormones such as cholecystokinin, gastrin, vasoactive intestinal peptide, thyrotropin-releasing hormone, calcitonin, corticotropin-releasing hormone, and vasopressin [186].

Biological significance of PAM is not fully understood, but deficiency results in widespread effects. Brindled mice, a genetic model of ATP7A-related copper disturbances, fail to produce normal levels of α-amidated peptides [198,199]. PAM deficient mice show CNS problems, e.g., impaired vasoconstriction and thermoregulation, increased seizure susceptibility, anxiety, and increased response to noise [198].

### 6.3. Monooxygenase, DBH-Like 1 (MOXD1)

Monooxygenase, DBH-like 1 (MOXD1) is structurally similar to other ascorbate requiring copper-containing monooxygenases, but with unknown substrate. MOXD1 lacks signal sequence and localizes throughout ER in both endocrine and non-endocrine cells [200]. MOXD1 is membrane-associated and oligomerize. MOXD1 is predicted to hydroxylate a substrate in ER, and possibly acts as enzyme chaperone for DBH [200].

### 6.4. Tyrosinase (TYR)

Tyrosinase (TYR) catalyzes the first two steps in melanogenesis from tyrosine to DOPA and to dopaquinone. Tyrosine oxidation is rate-limiting followed by ER polymerization reactions [201,202] catalyzed by two members of tyrosinase-related proteins TYRP1 and TYRP2 [203].

TYR is membrane anchored and possesses two copper centers resembling DBH though entirely made up of His [185,204]. Copper is acquired during maturation in secretory pathway, but apo-TYR can be activated later by addition of copper [205,206]. TYR localizes to specialized endosomes termed melanosomes and undergoes maturation and sorting before reaching integration site [207,208]. Intracellular sorting and polymerization steps from ER through Golgi to melanosomes is tightly regulated including metalation and N-glycosylation [203,209,210]. During sorting in ER, TYR interacts with lectins normally associated with LMAN1 [207,209]. Metalation likely occurs in ER before action of TYRP1 and TYRP2, but can take place later in melanosomes, and TYR becomes fully functional only at its final destination [210,211]. TYRP1 and TYRP2 belong to the same protein family and have similar metal binding sites though using zinc. TYR substrates play a conformational role as molecular chaperones to enhance folding and ERGIC trafficking [208]. The Met-His-rich first luminal loop of ATP7A possibly metalates TYR [28]. TYR may lose copper during passage of acidic TGN but is reloaded in melanosomes with neutral pH [210,212].

Melanosomes originate from distinct, though related, embryonic stem cells: (1) neural tube derived retinal pigment epithelium and pineal gland melanocytes; (2) neural crest derived melanocytes of inner ear, skin, hair-bulbs, and iris [213]. Highest TYR expression is in pigment epithelium of retina. Skin melanosomes are transferred to keratinocytes where melanins protect against UV sun radiation [214]. Melanins are negatively charged, polymerized and hydrophobic pigments working as capacitor to absorb and dissipate energy to neutralize radiation. In case of high energy absorption, output occurs as heat and reactive oxygen species (ROS), eventually resulting in sun burn and necrosis. Complex neuromelanins are synthesized mainly in dopaminergic neurons of substantia nigra and noradrenergic neurons of locus coeruleus [215]. Midbrain catecholaminergic neurons of basal ganglia network are crucial for brain cognitive functions. Biosynthesis and regulation of neuromelanins are poorly understood [216,217] as is their role in smell, vision, and hearing. Deficient development of inner ear melanocytes causes deafness [218,219]. TYR mutations result in hypopigmentation disorders and sensitivity to UV radiation, visual problems like nystagmus, strabismus, and reduced visual acuity with photophobia [220]. Transduction overload may lead to local oxidative stress and accumulation of waste products in central and peripheral ganglions and increased risk of melanoma [217]. Pigment and cell debris accumulation in CNS may increase susceptibility to Parkinson [217].

In MNK visual problems are early onset nystagmus, iris trans-luminescence, hypo-pigmented fundus, and reduced visual acuity [221]. Hearing may be impaired, but often not investigated. In accord with above, copper replacement therapy in MNK shows darkening of hair and skin [206].

## 7. Copper/Zinc-Containing Superoxide Dismutases (Cu/Zn-SODs)

Superoxides are products of normal aerobic metabolism and crucial in oxidative burst of innate immune responses [222], but in need of strict control. Superoxide dismutase (SOD) disproportionate the reactive radicals into molecular oxygen and less reactive hydrogen peroxide. Uncontrolled, ROS will attack unsaturated fatty acids, and SOD is of particular importance for a healthy brain, and of the most abundant enzymes underlining importance of ROS control. SOD1 is compartmentalized into distinct cellular and minor extracellular pools. SOD3 is attached to extracellular matrix, and often named extracellular SOD (EC-SOD). SOD1 activity is copper regulated at protein level, while SOD3 activity is copper regulated at gene level. Amyloid-β precursor protein (APP) family consists of Cu/Zn proteins with a SOD-like structure and possible dismutase activity [223] and is copper regulated through Cu-CCS activated cleavage of β-secretase 1 (BACE1). A manganese form (SOD2) in mitochondrial matrix is interconnected with IMS-SOD1 [224] and SOD3 [225].

### 7.1. Superoxide Dismutase 1 (SOD1)

Superoxide dismutase 1 (SOD1) is the master SOD and sole cytosolic and peroxisomal cuproenzyme. SOD1 mainly localizes in cytosol, an almost equal fraction in peroxisomes [226], and minor pools in mitochondrial intermembrane space (IMS), and nucleus. SOD1 comprise a large copper pool, earlier viewed as copper buffer [227], substantiated by labile metal binding by a cluster of four imidazole groups [11]. Some cell types secrete SOD1 [228]. SOD1 is unusual by having a labile copper site in cytoplasm abundant in GSH, and likely needs shielding by vesicular structures [229]. Copper chaperone for SOD1 (CCS) participates in maturation and activation of SOD1 at all subcellular locations. CCS is a member of the Cu/Zn-SOD family and acts as an enzyme chaperone to catalyze an intramolecular disulfide bond, stabilizing correct SOD1 conformation for incorporation of copper and zinc. CCS also functions as molecular chaperone and contains three domains having different roles: N-terminus possesses a copper binding site (MXCXXC), similar to ATOX1 [230], also with potential allosteric role in copper activation. The homologous middle part interacts with SOD1, and C-terminus contains a copper catalytic CXC site needed for intramolecular S-S bridge formation [231,232]. Nascent SOD1 and CCS polypeptides devoid of metal enter IMS individually and with essential sulfides reduced while traversing outer mitochondrial membrane [93,233]. In IMS, SOD1 meets CCS, and is folded and activated as in cytosol, hereby retained in IMS as functional enzyme. SOD1 and CCS are both taken up by the CHCHD4 (~MIA40) redox import machinery [234].

Peroxisomes enclosed by a single lipid bilayer use special import of membrane proteins and matrix enzymes [235]. Most contains a peroxisomal targeting signal (PTS) and are taken up via peroxin (PEX) membrane receptors [236,237] and delivered through direct contact between ER and peroxisomes. Folded, co-factor bound, and oligomeric proteins can be imported [238]. A major SOD1 route through ER has been discovered, securing high peroxisomal matrix content [239]. SOD1 does not contain PTS and is piggy-backed into peroxisomes by its chaperone [237,239]. CCS-PTS is in ER recognized by PEX5 receptor, shuttling CCS-SOD1 into peroxisomal matrix [235]. SOD1 rapidly enters nucleus in response to increased H_2_O_2_ levels and is potentially piggy-backed via ER by CCS. Peroxisomes are present in all tissues catalyzing a wide range of anabolic and catabolic reactions. SOD1 generates H_2_O_2_, and catalase uses H_2_O_2_ to oxidize substrates. SOD1 dysfunction leads to ROS accumulation that eventually damage the peroxisomal membrane, and release catalase to cytosol [240]. Severe pathologies result from peroxisomal dysfunction showing multi-systemic symptoms referred to as peroxisome biogenesis disorders (PBD). Neurological dysfunction is prominent usually accompanied by brain malformations, myelin abnormalities, and neuronal degeneration [241]. Systemic manifestations often include dysmorphic features, liver dysfunction, and skeletal abnormalities [241].

In MNK brain, both CCS and SOD1 polypeptides are taken up into mitochondrial IMS, but SOD1 is not properly folded and activated due to lack of copper. ROS are expectedly high, and matrix SOD2 induced as compensation [224]. The ER-peroxisomal route is also compromised creating a deficit of peroxisomal matrix SOD1 and enhanced peroxisomal stress in turn affecting nerve development. Still CCS accumulates [224,242] indicating faulty heterodimerization when copper is low. Deficiencies affect cerebellar maturation and axonal integrity, and lead to Purkinje cell pathologies with “weeping willow”, a well-recognized sign in MNK [242,243]. Low hepatic copper results in low SOD1, and oxidative stress plays a role in the pathogenesis of steatosis.

If nascent SOD1 is not correctly processed, it will remain inactive, potentially misfold, dimerize or tetramerize, as is the case in some neurodegenerative diseases [244]. Genetic disturbances of SOD1 lead to motor neuron disease, amyotrophic lateral sclerosis (ALS). Most SOD1 mutations affects heterodimerization and piggy-backing into peroxisomes [245]. Like MNK, lack of peroxisomal uptake of SOD1 will in ALS lead to oxidative stress and development of varying motor neuron affection as part of the PBD spectrum.

### 7.2. Superoxide Dismutase 3 (SOD3)

Extracellular superoxide dismutase (SOD3) is anchored to heparan sulfate in ECM [246]. SOD3 is structurally closely related to SOD1 and also contains copper in its catalytic center and zinc to stabilize structure. The central part of SOD3 is homologous to SOD1, the metal binding sites preserved and with similar copper avidity, but structures vary at ends. SOD3 contains a signal peptide plus three N-glycosylation sites for GAG guidance [247]. The enzyme is copper loaded in secretory pathway, but no specific copper chaperone has been identified. ATOX1 regulates protein expression through copper dependent binding to SOD3 promoter [248]. C-terminus contains a heparan binding domain securing attachment to ECM [246]. After secretion SOD3 forms tetramers stabilized by intermolecular disulfide bonds. SOD3 is secreted by fibroblasts and glial cells and protects cell membranes against ROS; about 1% is free in plasma, lymph, and cerebrospinal fluid [249]. SOD3 levels are high in vasculature, heart, lungs, kidney, and placenta [250]. Low SOD3 activity is linked to lung disease such as acute respiratory distress syndrome or chronic obstructive pulmonary disease [251] and deficiency may result in angiopathy.

## 8. Cu/Zn-SOD-Related Proteins Regulated by β-Secretase 1 (BACE1)

Amyloid-β precursor protein (APP), and amyloid-like proteins APLP1 and APLP2 contain a dismutase fold resembling Cu/Zn-dismutases and are regulated by copper through protease cleavage [11,224]. They bind copper and zinc primarily through His coordination [252] but an enzymatic role has not been established, though APP redox capacity has been demonstrated [253,254]. APP and its processed forms appear to have a growth-factor-like role and promotes neuronal proliferation and division [255]. Thus, the APP family is important for synaptic development and plasticity of central and peripheral nervous systems [256]. We will point to the relationship between the APP family and the Cu/Zn-SOD family to emphasize remote regulation by CCS-Cu.

β-secretase 1 (BACE1) is a membrane-bound protease, catalyzing first step of extracellular release of soluble amyloid β peptide (Abeta) from APP. BACE1 is rate-limiting in neuronal Abeta generation and also cleaves numerous other substrates important in formation of myelin. BACE1 contains a CCS-Cu regulatory site spatially separated from the protease site [5,11]. N-terminal CCS-MXCXXC binds to a cysteine rich area in C-terminal cytoplasmic tail of BACE1 regulating numerous brain functions including PAM [257]. BACE1 is expressed at high levels in brain and pancreas. Expression is highest in substantia nigra, locus coeruleus and medulla oblongata [258]. Abrogated cleavage in BACE1 knockout mice shows a role in neuronal migration, axonal growth, and muscle spindle function [259]. BACE1 is N-glycosylated [260] implying poor ERGIC trafficking in addition to poor protease activity secondary to low brain copper in MNK.

## 9. Conclusions

The main objective of this review is tying enzymes, substrates, and key symptoms together in a unified hypothesis to explain Menkes disease symptoms and pathologies (Table 3). We also wish to shed light on crucial steps in biogenesis of copper-dependent enzymes (dysfunctional in MNK) by focusing on metalation sites in cells, metal chaperoning and trafficking of enzymes in the secretory pathway.

ATP7A disturbances result in complicated copper disorders starting by poor uptake at intestinal brush border, aggravated by poor release from enterocytes, further affecting all barriers in the body, underlining that the basic defect is not a simple copper insufficiency. Defects in reduction (STEAP) before cellular uptake and in oxidation (HEPH) before release contribute to a complex copper transport defect resulting in complex clinical traits. Intracellular organelle deficiencies develop, combined with copper accumulation in unavailable pools. Copper pumping into secretory pathway and enzyme metalation are clinically significant, and ERGIC enzyme trafficking is also emerging as a copper regulated step (LOX). MNK diagnosis is often missed until hair changes are obvious, and the delay may leave many undiagnosed cases. To improve diagnostic awareness, focus should be shifted from hair as the main diagnostic pointer to more subtle symptoms. We found no evidence of a copper specific sulfhydryl oxidase, and hair and skin changes likely result from combined lack of steroid sulfatase (SUMF1), copper amine oxidase (AOC), and defective mitochondrial Fe-S biogenesis. SUMF1 is a new player in Menkes disease linking faulty cholesterol biology to the clinical picture and a whole new group of GAG sulfatases, which may lead to mimicry of lysosomal storage disorders (Table 3).

Symptoms secondary to LOX dysfunction have been expanded and shed light on their role in activation of receptor and adapter collectin molecules. Though important, it is an overlooked component of Menkes disease pathology. In liver, LMAN1 deficiency affects coagulation factors V+VIII and alpha-1-antitrypsin, and in brain leads to poor trafficking of numerous neuroreceptors explaining nervous symptoms in MNK. Other receptors with a collagen-like stretch, COLQ and CCBE1, explain muscle weakness and lymphedema. Cq1 deficiency add problems with innate immunity, and lung surfactant defects. Unexpectedly the peroxisomal SOD1 pool requires ER for metalation, and Zellweger-like symptoms are becoming part of the MNK symptom spectrum. Interestingly, motor neuron disease is a characteristic of the mildest disease form, SMAX3.

Trafficking and post-translational modifications of copper enzymes, including metalation begin in the endoplasmic reticulum (ER) and continues in Golgi before proteins are sorted and sent to their final destinations. Sugar tags guide enzymes during folding, proof-reading, refolding, and holoenzyme trafficking [261]. In ER nascent polypeptides are core glycosylated, and the added sugar tags are used for cargo receptor recognition by LMAN1 and other lectins. Adaptor sugar recognition is important for correct folding and trafficking, and GAG defects lead to multiple tissue and organ failures as well as abnormal physiognomy. Proteins with a CRD domain constitute a distinct class of adaptor molecules of which collectins [109] require LOX for correct conformation and stability.

At present, the extent of glycosylation and trafficking defects in Menkes disease is unclear, and coppers significance for sugar sorting is an emerging field. LMAN1 is one of several homologous mannose binding adapter molecules securing protein trafficking in the secretory pathway. Further glycosylation modifications occur in the Golgi complex where an array of enzymes modifies the sugar tags for their final destination but may require metalation to expose N-glycosylation sites correctly [261]. Lack of copper can result in distorted conformation and lead to normally unexposed N-glycosylation sites being exposed or the opposite, resulting in integration at wrong membrane sites [261].

Copper metalation is most often cited as taking place in TGN, but we found clear evidence in the literature of metalation in ER. Possibly ATP7A delivers copper in ER, in ERGIC, and in Golgi. SUMF1 is resident and metalated in ER and requires an ER-resident homologue devoid of copper, SUMF2 as molecular chaperone. Strong indication exists that TYR and DBH are metalated in ER, also making ER-metalation of PHM likely. TYR sites have low avidity and if pH is low, often loses copper, but is reloaded in melanocytes with a neutral pH. Potentially ATP7A also provides copper here. TYR uses related molecules, TYRP1 and TYRP2 as molecular chaperones. TYRP1 and TYRP2 contain zinc and are both ER located. DBH is suggested to use the ER-resident homologue, MOX1D as molecular chaperone.

Blue copper oxidases (CP, HEPH, and HEPHL1) appear to be metalated in cis-Golgi. Cofactor formation of LOX and AOC happens by use of a redox process, before N-glycosylation in Golgi, though the process is normally cited as autocatalytic. However, required reactions will likely not rely on chance, but is facilitated by an enzyme reaction. HEPHL1 is needed for metalation of LOX, and AOC likely use the same or a similar redox chaperone. 

SOD1 is metalated at several cellular sites and depends on ER for metalation of the peroxisomal pool. CCS does not load SOD1 but is needed as redox chaperone to form S-S bridges stabilizing the conformation for proper metalation and subsequent piggy-backing of the CCS-SOD1 complex to peroxisomes. CCS provides allosteric regulation of SOD1 and BACE1, similarly to ATOX1 that allosterically regulates MBDs to initiate ATP7A/B pump activity.

ATP7A contains a Golgi localization signal and locates in ER when the signal is removed by alternative splicing. The first lumenal loop may help retain the protein in ER by binding of copper to Met-His-rich sequences similar to calcium ATPases using corresponding sites for calcium regulated ER retention.Metalation of DBH, PAM, and TYR may be facilitated by the Met-His-rich lumenal loop of ATP7A.

At experimental tissue culture conditions, ATP7A is found in TGN. However, most tissue culture experiments use fibroblasts, and the principal enzyme in this cell type is LOX, which is metalated in the late secretory pathway. Fet3 models will misinterpret ER activity as it is a CP/HEPH homologue metalated in Golgi. Thus, experiments using tissue culture may not represent the full picture of what takes place in vivo. We hypothesize that if ER metalation is diminished, all enzymes including downstream metalated enzymes may be affected leading to the severest phenotype. Milder phenotypes may preserve ER metalation of enzymes but show Golgi metalation problems. However, enzymes with low copper avidity may lose the metal during Golgi passage, and the enzyme may integrate at a faulty site resulting in deficient function, as may be the case for, e.g., DBH and TYR. Notably, all ATP7A-related phenotypes except SMAX3 show pale skin color and dysautonomia.

## 10. Future Directions

Numerous copper chaperones and adaptor molecules regulate copper-dependent enzyme passage in the secretory pathway, but the specific guiding molecules are only known for a fraction of copper enzymes. We hypothesize that more copper-dependent enzymes need specific copper chaperones for metal activation, and chaperoning roles may emerge for copper binding proteins for which today there is no known function. For example, the APP family may act as redox-active copper chaperones similarly to CCS. More copper-dependent enzyme reactions are likely to be unraveled, e.g., mitochondrial enzymes controlled by lipoic acid may also depend on copper. Finally, the iron–copper connection needs to be further explored on molecular, cellular, and organelle levels. Despite the new cellular/molecular connections outlined here for copper-dependent processes, the Menkes disease enzyme puzzle, linking consequences of ATP7A dysfunction in cells and tissue to MNK patients’ clinical symptoms, is not yet complete.

## Figures and Tables

**Table 1:** Clinical Phenotypes of ATP7A-linked Disorders.

Clinical Type *	Abreviation	OMIM	Age of Onset	Diagnostic Pointer	Connective Tissue Involvement	Motor Function *	Mental Function *	Age of Death ^§^
**Menkes Disease**	MNK	309400	0–8 months	Kinky Hair; floppy infant; mimicry of severe metabolic disorders of lysosomes, mitochondria, and peroxisomes; dysautonomia	Severe; osteoporoses; hemorrhages; bladder and bowel diverticulae	Poor mobility; no head control	Severe mental retardation	<3 years
**Long Surviving Menkes**	LS	309400	0–8 months	Hair changes; dysautonomia; floppy; initial symptoms similar to MNK	Severe to moderate	Poor mobility; limited head control	Severe mental retardation	<15 years
**Moderate Menkes**	MOD	309400	3–12 months	Coarse hair; dysautonomia; initial course milder; clinical diagnosis difficult	Present, but not obvious; skeletal dysplasia may occur	Wheelchair bound; cannot sit unsupported	Mentally retarded	<40 years
**Mild Menkes**	MILD	309400	2–3 years	Coarse hair; dysautonomia; initial course mild; clinical diagnosis difficult	Minimal to moderate; skeletal dysplasia;	Walk with difficulty and with use of aid	Moderately retarded; Slow and dull	>40 years
**Occipital Horn Syndrome ^#^**	OHS	304150	Childhood	Coarse hair; X-linked family history; connective tissue problems; muscle affection; dysautonomia,	Exostoses on occipital bones; skeletal dysplasia; cutis laxa; hyperextensible joints; vascular complications	Walk independently; appear clumsy	Slow or dull to normal IQ	40–60 years
**X-linked distal spinal muscular atrophy 3**	SMAX3	300489	Adulthood	X-linked family history of muscle wasting; dysautonomia with prevalent adrenergic involvement	Minor connective tissue involvement; minor occipital horns may occur	Walk independently; progressive distal motor neuropathy	Normal IQ; normal fertility	<80 years

* clinical types are poorly delineated with overlapping symptoms and functions within and between groups; ^#^ occipital horns depend on head control and can occur from two years; ^§^ connective tissue complications shorten lifespan; Cu treatment prolongs lifespan.

**Table 2:** Copper-dependent Enzymes and basic properties.

	Enzyme	OMIM	EC-no.	Cofactor	Cu Donor	Cu Loading Site	Cu Chaperone	Subcellular Localization	ATP7A-Linked Cu Deficiency Symptoms
**ATOX1 regulated** **Copper Pumps**	ATP7A	300011	7.2.2.8	Mg; ATP Cu-ATOX1 allosteric	ATOX1 Cu-GSH	Cytosol	ATOX1 piggy-backing	SP, TGN, PM	Cu storage in tissues, low in brain, liver, plasma; S-Cu diagnostic after 1.5 mo
	ATP7B	606882	7.2.2.8	Mg; ATP Cu-ATOX1 allosteric	ATOX1 Cu-GSH	Cytosol	ATOX1 piggy-backing	SP, TGN, secretory vesicles	Low activity in brain and liver; Fe accumulation; icterus, steatosis
**Copper Reductases**	STEAP1	604415	1.16.1.-	Heme; NAD	Cu-His	EC redox	NAp	PM, endosomes	Cu and Fe accumulation on plasma membranes and vesicles; hypochromic anemia
	STEAP2	605094	1.16.1.-	Heme; NAD	Cu-His	EC redox	NAp	PM, Golgi	
	STEAP3	609671	1.16.1.-	Heme; NADP	Cu-His	EC redox	NAp	PM, endosomes	
	STEAP4	611098	1.16.1.-	Heme; NADPH	Cu-His	EC redox	NAp	PM, ER, Golgi endosomes nucleus, MIT	
	CYBDR	605745	1.-.-.-	Heme; ascorbate	Cu-His	EC redox	NAp	PM	
**Copper Oxidases**	CP	117700	1.16.3.1	Cu; ascorbate	ATP7B	ERGIC	NK	EC	CP low in plasma; diagnostic after 1½ month; Cu and Fe storage
	FV+VIII	612309 300841	1.16.3.1	Cu; ascorbate	ATP7B	ERGIC	NK	EC	Mild clotting deficiency
	HEPH	300167	1.16.3.1	Cu; ascorbate	ATP7A	ERGIC	NK	Vesicles	Cu and Fe intracellular storage; AMD
	HEPH1L	618455	1.16.3.1	Cu; ascorbate	ATP7A	ERGIC	NK	*cis*-Golgi	Cutis laxa
	COX CuA (II) CuB (I)	516040 516030	1.9.3.1	Cu Cu;Heme	Redox SCO1; SCO2; COA6 COX11	IMS IMS	COX17 COX17	IMM	Low COX in brain and liver due to low Cu availability; High lactate in blood; Leigh-like symptoms; COX defects; ragged red fibers; hypotonia
**Copper Quinone Amine Oxidases**	LOX	153455	1.4.3.1	Cu; LTQ	ATP7A	*cis*-Golgi	HEPH1L redox	EC	Numerous connective tissue abnormalities: tortuous vessels, aortic aneurisms, and dissections, umbilical or inguinal hernias, bladder and bowel diverticulae, loose joint and skin, osteoporosis, lymphedema, lung infections; collectin defects with protein trafficking problems, and deficient activation of complement pathway; NAI-like; cataract
	LOXL1	153456	1.4.3.1	Cu; LTQ	ATP7A	*cis*-Golgi	Redox loading ^#^	EC	
	LOXL2	606663	1.4.3.1	Cu; LTQ	ATP7A	*cis*-Golgi	Redox loading ^#^	ER, nucleus *	
	LOXL3	607163	1.4.3.1	Cu; LTQ	ATP7A	*cis*-Golgi	Redox loading ^#^	ER, nucleus *	
	LOXL4	607318	1.4.3.1	Cu; LTQ	ATP7A	*cis*-Golgi	Redox loading ^#^	EC	
	AOC1	104610	1.4.3.22	Cu; TPQ	ATP7A	*cis*-Golgi	Redox loading ^#^	EC	Ichthyosis, alopecia, inflammation, conjunctivitis, atopy, photophobia, keratitis, diarrhoea, gastrointestinal polyps
	AOC2	602268	1.4.3.21	Cu; TPQ	ATP7A	*cis*-Golgi	Redox loading ^#^	PM	
	AOC3	603735	1.4.3.21	Cu; TPQ	ATP7A	*cis*-Golgi	Redox loading ^#^	PM	
**FGly** **Generation**	SUMF1	607939	1.8.3.7	Cu; Ca	ATP7A	ER	SUMF2	SP	GAG accumulation in tissues and urine; metachromasia, Alder Reilly anomaly; overlapping clinical features of multiple sulfatase deficiency (MSD) mimicking metachromatic leukodystrophy, mucopolysaccharidosis (MPS), mucolipidosis (MLP), chondrodysplasia punctata, hydrocephalus
**FGly** **Activated Sulfatases**	ARSA	607574	3.1.6.8	FGly; Ca	NAp	NAp	NAp	Lysosomes	
	ARSB	611542	3.1.6.12	FGly; Ca	NAp	NAp	NAp	Lysosomes	
	ARSD	300002	3.1.6.-	FGly; Ca	NAp	NAp	NAp	Lysosomes	
	ARSF	300003	3.1.6.-	FGly; Ca	NAp	NAp	NAp	EC	
	ARSE	300180	3.1.6.-	FGly; Ca	NAp	NAp	NAp	Golgi	
	ARSG	610008	3.1.6.1	FGly; Ca	NAp	NAp	NAp	Lysosomes	
	ARSH	300586	3.1.6.-	FGly; Ca	NAp	NAp	NAp	PM	
	ARSI	610009	3.1.6.-	FGly; Ca	NAp	NAp	NAp	EC	
	ARSJ	610010	3.1.6.-	FGly; Ca	NAp	NAp	NAp	EC	
	ARSK	610011	3.1.6.-	FGly; Ca	NAp	NAp	NAp	EC	
	GALNS	612222	3.1.6.4	FGly; Ca	NAp	NAp	NAp	Lysosomes	
	GNS	607664	3.1.6.14	FGly; Ca	NAp	NAp	NAp	Lysosomes	
	IDS	300823	3.1.6.13	FGly; Ca	NAp	NAp	NAp	Lysosomes	
	STS	300747	3.1.6.2	FGly; Ca	NAp	NAp	NAp	ER	ichthyosis, seborrhoea, hair changes
**Copper Amine Oxidases**	DBH	609312	1.14.17.1	Cu; ascorbate	ATP7A	ER	MOXD1^#^	Vesicles; EC	High DA/NE; vomiting, hypotension, hypothermia, hypoglycemia
	PAM PHM PAL	170270	1.14.17.3 4.3.2.5	Cu; ascorbate Zn; Ca	ATP7A NAp	TGN NAp	NK NAp	Golgi Vesicles	Pain, seizure, anxiety, impaired wakening, temperature, weight and fluid balance
	TYR	606933	1.14.18.1	Cu; ascorbate	ATP7A	ER	TYRP1 TYRP2	Melanosomes	Albinism, visual and hearing problems
	MOXD1	609000	1.14.17.-	Cu; ascorbate	ATP7A	ER	-	ER	~DBH deficiency
**Cu/Zn Superoxide Dismutases**	SOD1	147450	1.15.1.1	Cu; Zn Cu-CCS allosteric	GSH ATP7A matrix NK	Cytosol ER IMS NK	CCS redox and piggy-backing CCS redox CCS	Cytosol peroxisomes IMS nucleus	Low SOD1 activity in nerve tissue and liver due to poor Cu availability; peroxisomal pathologies; motor neuron disease
	SOD3	185490	1.15.1.1	Cu; Zn	ATP7A	SP	NK	EC	Lung disease, angiopathy
	CCS	603864	NA	Cu;Zn	GSH ATP7A matrix nucleus	Cytosol ER IMS nucleus	NAp	Cytosol peroxisomes IMS nucleus	Purkinje cell pathologies; ALS-like phenotype
	APP	104760	NA	Cu; Zn	NK	SP	NK	EC	Senecense, cerebral angiopathy
	APLP1	104775	NA	Cu; Zn	NK	SP	NK	EC	
	APLP2	104776	NA	Cu; Zn	NK	SP	NK	EC	
**Cu-CCS** **Regulated Enzyme**	BACE1	604252	3.4.23.46	Cu-CCS allosteric	CCS	SP	CCS	TGN	Poor neuronal growth

^#^: indicated; *: histone biology; EC: extracellular; ER: endoplasmic reticulum; ERGIC: ER–Golgi intermediate compartment; GAG: glycoamino glycans; GHS: glutathione; IMM: inner mitochondrial membrane; IMS: intra mitochondrial space; MIT: mitochondria; NA: not assigned; NAI: non-accidental injuries; NAp: not applicable; NK: not known; PM: plasma membrane; SP: secretory pathway; TGN: *trans*-Golgi network.

**Table 3:** Menkes Disease Symptoms.

Birth and Neonatal Period	Enzyme Deficiency	Comments
Preterm	AOC (histaminase)	One-third before 37 weeks
Premature rupture of fetal membranes	LOX	
Weight	-	One-third less than 2500 g
Length	-	
Head circumference	-	
Apgar score	-	Quick test at 1 and 5 min, in rare cases, also 10 min after birth
Denver scale	-	Developmental score for milestones in young children according to age
Bayley score	-	Cognitive, language, and motor developmental infants and toddlers score
Hydrops fetalis	LOX	Severe swelling (oedema)
Intrauterine growth retardation	LOX, SUMF1	Small for gestational age
Decreased fetal movements	-	
Neonatal onset	-	Rarely recognized before hair changes at 2–3 month
Neonatal death	-	
Early death	-	Usually before three years
Failure to thrive	-	
Feeding difficulties	DBH, LOX	Poor sucking and swallowing
Floppy infant	COX, LOX	
Poor head control	COX	
Dysautonomy	DBH	
Infantile spasms	COX	Shivers or a small jerks in series
Irritability	DBH, PAM, SUMF1	
Babinski reflex	DBH, PAM, SUMF1	Upward movement of the big toe sign of pyramidal dysfunction
Anxiety	DBH, PAM, SUMF1	
Increased response to noise	DBH, PAM, SUMF1	
Lethargy	DBH, PAM, SUMF1	Decreased alertness
Respiratory distress	LOX, SOD3	
Icterus/jaundice	CP, LOX	Photo therapy resistant
**External features**		
**- Head and neck**		
Face lacking in expression	DBH	Low mimic
Pallor	TYR	Light skin color
Hypertelorism	LOX, SUMF1	Widely spaced eyes
Nystagmus	DBH, LOX, TYR	Difficulty in controlling eye movements
Blepharophimosis	LOX	Narrowing of eye opening
Photophobia	AOC, TYR	Light intolerance
Keratitis	AOC, LOX	Cornea inflammation
Conjunctivitis	AOC, LOX	Eye inflammation
Ptosis	DBH, LOX	Droopy eyelids
Miosis	DBH	Excessive constriction of pupils
High arched (cupid) eyebrows	LOX, SUMF1	
Ophthalmoplegia	DBH, LOX	
Cherubic appearance	LOX, SUMF1	
Microcephaly	LOX, SUMF1	<2 SD for age
Brachycephaly	LOX, SUMF1	
Frontal bossing	LOX, SUMF1	
Occipital bossing	LOX, SUMF1	
Long philtrum	LOX, SUMF1	
High forehead	LOX, SUMF1	
High-arched palate	LOX, SUMF1	
Small chin	LOX, SUMF1	
Pudgy cheeks	SUMF1	
Flat central face	LOX, SUMF1	
Depressed nasal bridge	LOX, SUMF1	
Nasal congestion	LOX	
Hypoplastic mandibles	LOX, SUMF1	
Micrognathia	LOX, SUMF1	
Retrognathia	LOX, SUMF1	
Drooping jaws	SUMF1	
Low set ears	LOX, SUMF1	
Large ears	LOX, SUMF1	
Occipital exostoses	LOX	Calcified exostoses palpable from occiput, uncommon
Internal jugular phlebectasia	LOX	
**- Chest**		
Pectus excavatum	LOX	
Pectus carinatum	LOX	
**Neurological symptoms**		
Corpus callosum agenesis	SUMF1	Absence of brain structure that connects the two hemispheres
Dysautonomia	DBH	
Cerebellar hypoplasia	LOX	
Mental retardation	COX, PAM, SOD1	
Motor retardation	COX, PAM, SOD1	
Loss of milestones	-	Progressive neurologic defects
Hypothermia	DBH, PAM	Subnormal body temperature
Hypoglycemia	DBH, PAM	Subnormal sugar values
Nasal congestion	DBH	
West syndrome	COX	Epileptic encephalopathy
Seizures	COX	Refractory and early onset
Clonic seizures	COX	
Myoclonic seizures	COX	
Tonic seizures	COX	
Motor dysfunction	DBH	
Ataxia	DBH, PAM, SOD1, SUMF1	
Spasticity	COX	
Hypertonia	DBH	
Hypotonia	DBH	
**Eye symptoms**		
Cataract	LOX	
Myopia	LOX	
Nystagmus	DBH, LOX, TYR	Difficulty in controlling eye movements
Strabismus	TYR	
Blepharophimosis	LOX	Narrowing of eye opening
Photophobia	AOC, TYR	Light intolerance
Keratitis	AOC, LOX	Cornea inflammation
Conjunctivitis	AOC, LOX	
Ptosis	DBH, LOX	Droopy eyelids
Miosis	DBH	Excessive constriction of pupils
Reduced visual acuity	TYR	
Optic discs palor	TYR	
Optic atrophy	TYR	Abnormal electroretinogram (ERG)
Visual loss	TYR	Visual evoked potential (VIP)
Retinal and iris depigmentation	TYR	
Iris trans-luminescence	TYR	
Iris microcysts	SUMF1, TYR	
Hypopigmented fundus	TYR	Fundoscopy
**Ear symptoms**		
Hearing loss	LOX, PAM, TYR	Brain stem auditory evoked potential (BAEP)
**Hair and skin symptoms**		
Fine, silvery and brittle hair	AOC, TYR, SUMF1	Short, stubby, friable
Depigmented scalp hair	TYR, SUMF1	Lusterless, silvery, steel wool
Sparse hair	AOC, SUMF1	Rubbing against pillow may feel like unshaven stubbles
Alopecia	AOC, SUMF1	Lack of hair
Fetal hair may be unaffected	-	Soft
Pili torti	SUMF1	Hair twisted about their own axis
Trichorhexis nodosa	SUMF1	Frying and splitting of hair ends
Monilethrix	SUMF1	Varying diameters of the shafts
Cupid eyebrows	LOX, SUMF1	Eyebrows with a high arch
Sparse eyebrows	AOC, SUMF1	Look like old man’s eyebrows
Sparse eyelashes	AOC, SUMF1	Breaks easily
Seborrhea	AOC, SUMF1	Dry and scaly skin
Erythroderma	AOC, SUMF1	Generalized exfoliative dermatitis with redness and scaling
Cutis laxa	HEPHL1, LOX	Lax and wrinkled skin may give a progeria like appearance
Pale skin	PAM, TYR	Almost like an albino
Anhydrosis	DBH, LOX	Inability to sweat normally
Doughy skin	LOX	Swelling of subcutaneous tissue
Lymphedema	LOX	Swelling due to poor lymphatic system
**Dentation**		
Hyperplastic gums	LOX	Prominent gums
Dental abnormalities	LOX	
Enamel defects	LOX	
Delayed eruption	LOX	
Biconically shaped incisors	LOX	
**Lung symptoms**		
Acute respiratory distress syndrome	AOC, LOX, SOD3, SUMF1	
Chronic obstructive pulmonary disease	AOC, LOX, SOD3, SUMF1	
Emphysema	LOX, SOD3, SUMF1	Damaged air sacs (alveoli) with breathing difficulty
**Cardiovascular symptoms**		
Congenital heart disease	COX, LOX	About 5%
Angiopathy	AOC, APP, LOX, SOD3	Disease of arteries, veins, and capillaries
Tortuous blood vessels	LOX	Twisted with frayed and split inner walls
Bleeding tendency	FV+VIII, LOX	
Mild coagulation deficiency	FV+VIII	
Hematomas	LOX	
Subdural hematomas	LOX	
Intracranial hemorrhage	LOX	
Cephalohematomas	LOX	Prevalent at birth
**Gastrointestinal symptoms**		
Chronic diarrhea	AOC	
Vomiting	AOC	
Bowel dysfunction	AOC	
Gastrointestinal polyps	LOX	
Hiatal hernia	LOX	
**Hepatic symptoms**		
Hepatomegaly	COX, SOD1, SUMF1	Low hepatic copper gives low enzymatic activity
Icterus	CP, ATP7B	Yellowish color of skin and eyes
Steatosis	COX, SOD1	Fatty liver
**Genitourinary symptoms**		
Bladder diverticula	LOX	
Bladder rupture	LOX	
Ureteral obstruction	LOX	
Glomerulonephritis	LOX	
Urinary tract infection	LOX	
Vesico-ureteral reflux	LOX	
Hydronephrosis	LOX	Partial urinary tract blockage
Diaphragmatic hernia	LOX	
Umbilical hernias	LOX	
Inguinal hernia	LOX	
Cryptorchidism	LOX, SUMF1	Undescended t esticles
**Connective tissue symptoms**		
Loose/hypermobile joints	LOX	
Tortuous vessels	LOX	
Wrinkled and loose/extensible skin	LOX	
Soft skin / edema	LOX	
**Musculoskeletal symptoms**		
**- Skeletal—neck and chest**		
Cervical spine anomalies	LOX	Mimics non-accidental lesions
Short, broad clavicles	LOX	
Flaring of the ribs	LOX	
Short, broad ribs	LOX	
Pectus excavatum	LOX	Sunken breastbone
Pectus carinatum	LOX	Protruding breastbone; “pigeon chest”
**- Skeletal—limbs**		
Congenital bone fractures	LOX	Symmetrical uncommon in “battered child”/NAI
Long-bone fractures	LOX	
Metaphyseal spurring	LOX	Can resemble scurvy
Diaphyseal periosteal reaction	LOX	
Cortical thickening	LOX	
Short humeri	LOX	
**- Skeletal—others**		
Wormian bones	LOX	Intrasutural supernumerary bones, not found in child abuse
Spondylolysis	LOX, SUMF1	Fractures of vertebra
Osteroporosis	LOX, SUMF1	Brittle bones
Osteopenia	LOX, SUMF1	
Cartilage malformation	LOX, SUMF1	
Joint laxity	LOX	
Limb dislocations	LOX	
Metaphyseal widening	LOX	
Osteochondrodysplasia	LOX, SUMF1	
Occipital horn exostoses	LOX	Uncommon in MNK, but can be observed from 2 years
**- Muscles**		
Motor neuron disease	SOD1, LOX	
**Investigations**	**Activity measured**	
MR MRI and MRA	Neuroimaging	Magnetic Resonance and computerized tomography; cerebral atrophy, cortical areas of low density, diffuse cerebral and cerebellar volume loss, white-matter, and basal ganglia changes
CT	Neuroimaging	
EEG	Brain activity	Hypsarrhythmia, diffuse, multifocal spike activity
Radiography	Bone	Symmetrical metaphyseal flaring and spurring of ribs, and cervical fractures may mimic non-accidental trauma, but these are not symmetrical; skull wormian bones are not seen in child abuse
Arteriography	Vasculature	Elongated and tortuous cerebral and systemic vessels
Ultrasonography	Bladder, bowel	Diverticulae and polyps
Light Microscopy	Hair examination	Pili torti, trichorexis nodosa, monilethrix
Echocardiography	Heart	Heart murmur
ERG	Electroretinogram	Optic atrophy
VIP	Visual evoked potential	Loss of vision; retinal and macular degeneration
Fundoscopy	Eye background, macula	Hypopigmented
BAEP	Brain stem auditory evoked potential	Hearing loss
Cell culture	Radioactive copper test	Increased accumulation and retention
Tissue copper	ICPMS; AA	Increased in CVS, placenta, muscle; liver low
**Biomarkers**		
Boy	ATP7A	X-linked
Family history of male infant death	ATP7A	X-linked
Hyperbilirubinemia	ATP7B	Transient, but prolonged and light therapy resistant
Low plasma copper	ATP7A	Diagnostic from 4–6 weeks
Low free Cu	ATP7A	Diagnostic from birth
Low ceruloplasmin	CP	Diagnostic from 4–6 weeks
High RBC (Red Blood Cells) Cu	SOD1	Erythrocyte SOD1 in neonates
Anemia	HEPH, CP	May be hypochrome
Neutropenia	LOX	Decreased neutrophils
Thromboembolism	FV+VIII	Blood clot breaking loose and plugs other vessels
Urinary Cu low to normal	ATP7A	MT
Low liver Cu	ATP7A	Diagnostic from birth
High placenta Cu	ATP7A	Diagnostic from birth; CVS diagnostic prenatally
High metallothionein levels	ATP7A	MT1 and MT2 (diagnostic?)
Plasma DA/NE ratio increased	DBH	Diagnostic from birth
Urinary HVA/VMA ratio increased	DBH	Diagnostic from birth
Hypoglycemia	SUMF1, PAM, DBH	Transient
High blood lactate	COX	CSF, intermittent
Pyruvate	COX	Intermittent
Hyperammoniemia	COX	Intermittent
High plasma glutamic acid	COX	Intermittent, alpha-ketogluterate conversion
Respiratory chain deficiencies	COX	Indicative
Intracellular Cu accumulation	ATP7A	Diagnostic, tissue culture
Molecular screening of *ATP7A*	ATP7A	Definitive diagnosis
**Pathology**		
Purkinje cell pathologies	SOD1	Faulty arborization and “weeping willow”
Ragged red fibers	COX	Subsarcolemmal aggregates of mitochondria in muscle fiber
Alder Reilly anomaly	SUMF1	Vacuolization of blood cells; observed in GAG deficiencies
Metachromasia	SUMF1	Color staining change of accumulated tissue sugar sulfatides
Pili torti	SUMF1	Hair twisted about their own axis
Trichorhexis nodosa	SUMF1	Frying and splitting of hair ends
Monilethrix	SUMF1	Varying diameters of the shafts
**Differential diagnosis:**		
NAI	LOX	Non-accidental injuries; >10% symmetric changes think MNK
Osteogenesis imperfecta	LOX	Brittle bones and bone dysplasias
Mitochondrial disorder	COX	Compromised energy production affecting all organs and tissues
Organic acid uria	COX	Defective mitochondrial matrix metabolism
Cutis laxa	LOX	Loose and wrinkled skin in an infant
Progeria	LOX, SUMF1	Old age syndrome in young people
Syndromes with hair abnormalities	SUMF1, AOC	
Glutamine defects	COX	Defective mitochondrial matrix metabolism
MSD	SUMF1	Multiple sulfatase deficiency
MPS	SUMF1	Mucopolysaccaridoses
MLP	SUMF1	Mucolipidoses
Leukodystrophy	SUMF1	E.g., metachromatic leukodystrophy
DBH deficiency, congenital	DBH	CNS Cu deficiency

## Data Availability

Not applicable.

## Abbreviations

ABP1	Amiloride-binding protein 1
ALS	Amyotrophic lateral sclerosis
AMD	Age-related macular degeneration
AOC	Copper-containing amine oxidases
APP	Amyloid-β precursor protein
APLP	Amyloid-like protein
ARS	Arylsulfatase
ATOX1	Antioxidant 1 copper chaperone
ATP	Adenosine triphosphate
ATP7A	Copper-transporting ATPases, α
ATP7B	Copper-transporting ATPases, β
BACE1	β-secretase 1
BBB	Blood–brain barrier
C	Cysteine
C1q	Complement Q1
CCBE1	Collagen and calcium binding EGF domains 1
CCS	Copper chaperone for SOD1
CHCHD	Coiled-coil-coiled-coil domain
CNS	Central Nervous System
COA6	COX Assembly Factor 6
COL	Collagen
COLQ	Collagen-like tail of endplate acetylcholinesterase
COX	Cytochrom c Oxidase
CP	Ceruloplasmin
CRD	Carbohydrate-recognition domain
CSF	Cerebrospinal Fluid
Cu	Copper
Cys	Cysteine
CYBRD1	Cytochrome b reductase 1
DA	Dopamine
DAO	Diamine Oxidase
DBH	Dopamine β-hydroxylase
DOPA	Dihydroxyphenylalanine
ECM	Extracellular Matrix
EC	Extracellular
ELN	Elastin
ER	Endoplasmic reticulum
ERGIC	ER–Golgi intermediate compartment
FAD	Flavin adenine dinucleotide
FV+VIII	Clotting factors V and VIII
Fe-S	Iron sulfur site
FGE	Formylglycine generating enzyme
FGly	Formylglycine
G	Glycine
GABA	Gamma aminobutyric acid
GAG	Glycosaminoglycan
GALNS	Galactosamine-6-sulfate sulfatase
Gly	Glycine
GMXCXXC	Amino acid sequence of ATP7A/B MBD
GNS	N-acetylglucosamine-6-sulfatase
GPI	Glycosylphosphatidylinositol
GSH	Glutathione
HEPH	Hephaestin
HEPHL1	Hephaestin-like protein 1
His	Histidine
H_2_O_2_	Hydrogen peroxide
IDS	Idurunate 2-sulfatase
IMM	Inner mitochondrial membrane
IMS	Inter-membrane space
LMAN	Lectin mannose binding
LOX	Lysyl oxidase
LOXL	Lysyl oxidase-like
LTQ	Lysine tyrosylquinone
M	Methionine
MNK	Menkes disease
MBD	Metal binding domain
MXCXXC	Amino acid sequence of CCS-MBD
Met	Methionine
MIA40	Mitochondrial IMS assembly 40
MND	Motor neuron disease
MLP	Mucolipidose
MPS	Mucopolysaccharidose
MSD	Multiple sulfatase deficiency
MOXD1	Monooxygenase, DBH-Like 1
NAD(P)H	Nicotinamide adenine dinucleotide phosphate
NE	Norepinephrine
NLS	Nuclear localization sequence
OHS	Occipital horn syndrome
OMIM	Online mendelian inheritance in man
PAL	Peptidyl–α-hydroxyglycine α-amidating lyase
PAM	Peptidyl α-amidating enzyme
PAO	Polyamine oxidase
PEX	Peroxin
PHM	Peptidylglycine α-hydroxylating monooxygenase
PTS	Peroxisomal targeting signal
ROS	Reactive oxygen species
SCO	Synthesis of COX
SMAX3	X-linked distal spinal muscular atrophy 3
SOD	Superoxide dismutase
SP	Secretory pathway
S-S	Disulfide bridge
STEAP	Six-transmembrane epithelial antigen of prostate
STS	Steroid sulfatase
SUMF	Sulfatase-modifying factor
TGN:	Trans Golgi Network
TM	Transmembrane
TPQ	Trihydroxyphenylalanine quinone
TYR	Tyrosinase
TYRP	Tyrosinase related protein
VAP-1	Vascular adhesion protein 1
PBD	Peroxisome biogenesis disorders
Zn	Zinc
